# Engineering CAR-T cells

**DOI:** 10.1186/s40364-017-0102-y

**Published:** 2017-06-24

**Authors:** Cheng Zhang, Jun Liu, Jiang F. Zhong, Xi Zhang

**Affiliations:** 1 Department of Hematology, Xinqiao Hospital, Third Military Medical University, Chongqing, 400037 People’s Republic of China; 2 Division of Periodontology, Diagnostic Sciences & Dental Hygiene, and Division of Biomedical Sciences, Herman Ostrow School of Dentistry, University of Southern California, Los Angeles, CA USA

**Keywords:** Chimeric antigen receptor redirected T cells, CAR-T cells, Structure, Evolution, Production, Viral vector

## Abstract

Chimeric antigen receptor redirected T cells (CAR-T cells) have achieved inspiring outcomes in patients with B cell malignancies, and are now being investigated in other hematologic malignancies and solid tumors. CAR-T cells are generated by the T cells from patients’ or donors’ blood. After the T cells are expanded and genetically modified, they are reinfused into the patients. However, many challenges still need to be resolved in order for this technology to gain widespread adoption. In this review, we first discuss the structure and evolution of chimeric antigen receptors. We then report on the tools used for production of CAR-T cells. Finally, we address the challenges posed by CAR-T cells.

## Background

Chimeric antigen receptors (CARs) are engineered receptors that can graft an arbitrary specificity onto an immune effector cell (T cell). CARs include three parts: an extracellular antigen recognition domain of the single-chain Fragment variant (scFv) derived from an antibody), a transmembrane domain and an intracellular T cell activation domain of CD3ζ [1]. CAR-T cell therapy is designed to redirect a patient’s or donor’s T cells to specifically target and destroy tumor cells. This approach shows great promise for haematologic malignancies and solid tumors without major histocompatibility complex restriction [2]. In this review, we first discuss the structure and evolution of CARs. We then report on the tools used for production of CAR-T cells. Finally, we address the challenges posed by CAR-T cells.

## Structure of CAR-T cells

**Fig. 1:**
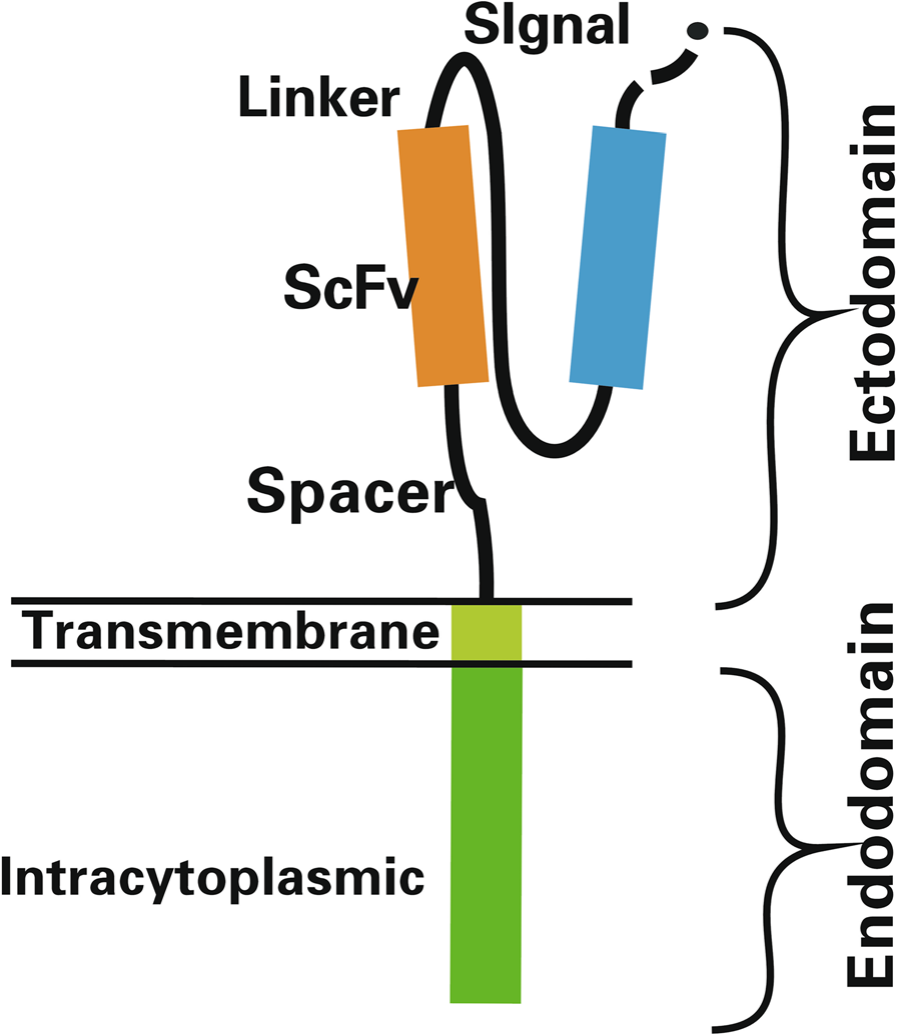
Structure of chimeric antigen receptor (CAR). The CAR includes ectodomain, transmembrane domain and endodomain

CARs consist of an ectodomain, transmembrane domain and endodomain [3] (Fig. 1).

### Ectodomain

The ectodomain is the domain of a membrane protein that is outside the cytoplasm and exposed to the extracellular space. The ectodomain in this case consists of signal peptide, antigen recognition region and spacer [1, 3].

The role of a signal peptide is to direct the nascent protein into the endoplasmic reticulum. The scFv, which serves as the signal peptide of the ectodomain in a CAR, is formed by the variable portions of heavy and light chains of an immunoglobulin fused through a flexible linker. An antigen recognition domain is usually a scFv with a simple ectodomain and more exotic recognition components, which can be used to recognize any antigen if it can bind targets with high affinity. The connection between the antigen binding domain and the transmembrane domain relied on the spacer. The simplest form of spacer is the hinge region of IgG1 and is sufficient for most scFv-based constructs.

### Transmembrane domain

The transmembrane domain, from the most membrane-proximal component of the endodomain, consists of a hydrophobic alpha helix that spans the membrane [1, 3, 4]. The stability of the receptor is related to the transmembrane domain. The presence of the native CD3-zeta transmembrane can cause the incorporation of the artificial TCR into the native TCR. At present, the CD28 transmembrane domain is the most stable receptor.

### Endodomain

The endodomain is the functional end of the receptor, and the most common component is CD3 ζ included three immunoreceptor tyrosine-based activation motifs (ITAMs) [3, 4]. After antigen recognition, the receptors cluster and signal was activated, then the sigal is transmitted to the T cell. Co-stimulatory signaling is needed during this profression.

## Evolution of CAR-T cells

**Fig. 2:**
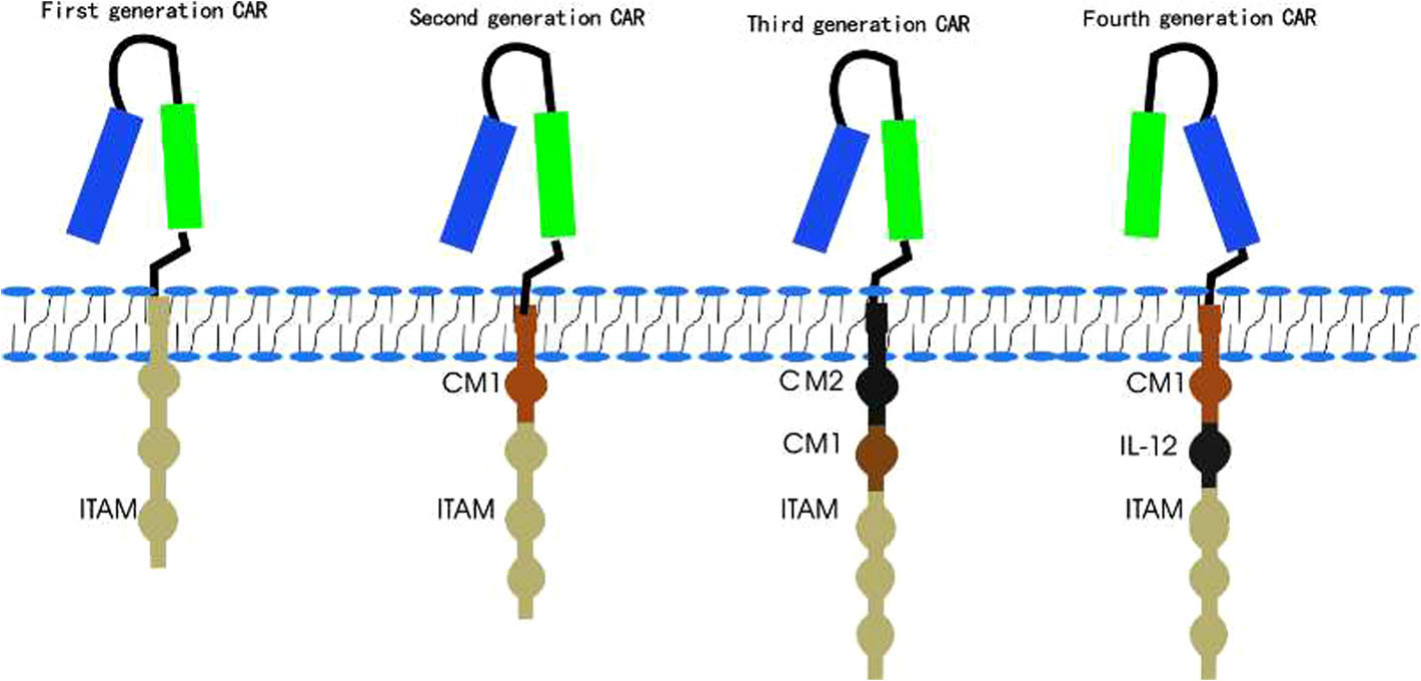
Evolution of chimeric antigen receptor (CAR) from the first generation to the fourth generation. Single chain antibody (CD3ζ or FcεRIγ) links the ITAM at transmembrane region for the first generation. Costimulatory molecule (CM1), such as CD28, has been engineered to the signal transduction region for the second generation. Another costimulatory molecule (CM2) based on the second generation for the third generation has been engineered to the signal transduction region, such as combining CD134 or CD137. The interleukin-12 (IL-12) based on the second generation for the fourth generation has been engineered to the signal transduction region. ITAM: Immunoreceptor tyrosine-based activation motifs

Since the initial development of CARs in 1989, CAR-T cells can be divided into four generations according to the structure of the endodomain (Fig. 2). The evolution of CAR therapy is an excellent example of the application of basic research to the clinic.

### First generation

Single structure from the CD3 ζ- chain or FcεRIγ from the intracellular domain for the first generation of CARs is the typical characteristics, which is the primary transmitter of signals from endogenous T cell receptor (TCR) [5, 6]. However, these CAR-T cells could not produce enough interleukin-2 (IL-2), so in order to kill tumor cells it was necessary to administer exogenous IL-2. Therefore, the first generation of CAR-T cell therapy transfected with single-chain receptors benefitted substantially from the accompanying administration of cytokines [7].

Recent studies showed that the deletion of phosphorylation on ITAM A and C in the CD3ζ signaling moiety could decrease the apoptosis signal, which is beneficial for the continuous expression of the transgene [8, 9]. However, more studies were performed with CAR-T cells with the CD3ζ-chain than the FcεRIγ-chain in clinics. The reason may be that three ITAMs were included in CD3ζ-chain, but only one in the FcεRIγ-chain. On the other hand, the CAR-T cells with the CD3ζ-chain were more effective at activating T cells and killing tumor cells, although they had lower expression levels *in vitro*. The transmembrane domain of CAR-T cells consists of a dimer of homologous or heterologous in combinations of CD3, CD8 and CD28, which can mediate optimal cellular activation via the dimerization of CARs and the functional interaction of this receptor with the endogenous TCR. Carcinoembryonic Ag-specific CD3ζ (MFEζ) -CAR-T cells, alpha-folate receptor (FR) -CAR-T cells, CE7R-CAR-T cells, scFv(G250)-CAR-T cells, GD2- CAR-Tcells and CD10- CAR-T cells were used to treat different tumors [10–15]. However, most of these studies with first-generation CAR-T cells did not achieve the desired outcomes because of inadequate proliferation, a short life span *in vivo* and insufficient secreted cytokines.

### Second generation

Dual signal was the typical characteristics for T cell activation. Three different receptor types including the T-cell antigen receptors, cytokine receptors and co-stimulatory receptors are included in this progression. The first signal is the special signal that, triggered by the TCR, recognizes the antigenic peptide-MHC complex on the surface of antigen-presenting cells. The second signal is the co-stimulatory signal, produced by a co-stimulatory molecule such as CD28/B7, which promotes the IL-2 synthesis to complete the activation of T cells and avoid apoptosis. Naïve T cells cannot perform their normal role if the co-stimulatory signal is absent, and the same is true even if the T cells are stimulated by the antigen. Therefore, CARs that only include the CD3ζ sequence cannot activate CAR-T cells without a co-stimulatory signal. Accordingly, second generation CARs added intracellular signaling domains from various co-stimulatory protein receptors to the cytoplasmic tail of the CARs to provide additional signals to the T cell, such as CD28 or CD137(4-1BB and CD134(OX40)), which can improve the proliferation, cytotoxicity, and sustained response, and prolong the life of CAR-T cells *in vivo* [16–18]. CD28-mediated co-stimulation is very important in the regulation of proliferation and survival for lymphocytes, and plays a key role for the establishment of memory cells and effector cells. CD134 can sustain proliferation and strengthen IL-2 production. CD137 can maintain the response signal of T cells, which plays a key role in the survival of T cells and the memory of CD8^+^ T cells [19–21]. The scFvCD19-CD137-CD3-CAR-T cells, MOv19-BBζ-CAR-T cells and scFvCD19-CD28-CD3ζ-CAR-T cells were used to treat B cell malignancies, and achieved better outcomes than the first generation [22, 23]. It seems that the 4-1BBζ-CAR-T cells have a longer persist time than CD28ζ-CAR-T cells, however, the direct comparisons is absent [24]. The CD28ζ-CAR-T cells can cause constitutively stimulation, proliferation and growth [25]. However, the 4-1BBζ-CAR-T cells can induce early exhaustion, which may limit antitumor efficacy [26, 27].

### Third generation

The third-generation CARs were made by combining multiple signaling domains, such as CD3ζ-CD28-OX40 or CD3ζ-CD28-41BB, to augment potency with stronger cytokine production and killing ability [28]. These scFv CD20-CD28-CD137-CD3ζ-CAR-T cells and HER2-CAR-T cells were used to treat lymphoma and colon cancer; however, outcomes were not improved relative to the second generation [29, 30]. The reason may be that the number of cases studied was small. Therefore, further studies are needed to explore the safety and efficacy of these treatments, and the selection of co-stimulatory molecules is also important.

### Fourth generation

The fourth-generation CARs were generated by adding IL-12 to the base of the second-generation constructs, and are known as T cell redirected for universal cytokine-mediated killing (TRUCKs). TRUCKs augment T-cell activation and activate and attract innate immune cells to eliminate antigen-negative cancer cells in the targeted lesion. It would be worthwhile to explore the role of TRUCKs in shaping the tumor environment by the inducible release of transgenic immune modifiers. Such TRUCK T cells can also treat viral infections, metabolic disorders and auto-immune diseases [31].

Altogether, these successive generations of CAR-T cell therapy have generated a great deal of enthusiasm in cancer treatment [32].

## Tools of transduction for CAR-T cells

A tool is needed for the delivery of the foreign gene into human cells. At present, there are two ways to accomplish gene incorporation with vectors, i.e., viral systems and non-viral systems.

The major vectors for gene therapy in basic research and clinical study are viruses, because of the high transfer efficiency, the relatively short time needed to reach the clinically necessary numbers of cultured T cells and the availability of different viruses with different expression characteristics. Most viral systems can accommodate genes from helpful and interesting cells and can provide the viral structural enzymes and proteins to allow for the generation of vector-containing infectious viral particles. The virus vectors include retroviruses (including lentivirus), adenovirus and adeno-associated virus. Among them, the most popular tools for gene delivery are genetically engineered retroviruses [33]. Retroviridae is a family of retroviruses. In this family, amino acid and nucleotide sequence, genome structure, pathogenicity and host range is difference. However, the virus vectors pose a potential safety hazard. The insertion mutation used to induce the immune reaction can lead to tumorigenesis and toxicity, the carrier capacity is limited and the titer achieved is not high enough [33, 34].

Non-viral gene therapy has maintained its position as an approach for treating cancer because of its higher efficiency, target specificity, non-infectiousness, unlimited carrier capacity, controlled chemical constitution and generous production, [35, 36]. Non-viral vectors include nude DNA, liposomes, polymerizers and molecular conjugates. Minicircle DNA vectors free of plasmid bacterial DNA sequences are novel non-viral vectors which are generated in bacteria from a parental plasmid, and can persistently express transgene with high levels *in vivo*. It is practicable in clinics for this method [37, 38].

## Production of CAR-T Cells

Several steps are required for the production of CAR-T cells. It is also very important to conduct quality control testing throughout the entire protocol [39].

First, leukocytes are taken from the patient’s or donor’s body using leukapheresis. Second, The T cells are enriched and washed to separate them from the leukocytes [40]. Third, the T cell subsets at the level of CD4/CD8 composition are separated using specific antibody bead conjugates or markers. Culture is then needed to activate the T cells. This process requires purifying autologous antigen-presenting cells (APCs) from the patients or donors, or beads coated with anti-CD3/anti-CD28 monoclonal antibodies, or anti-CD3 antibodies alone or in combination with feeder cells and growth factors, such as IL-2. IL-2 is the most commonly used agent because it induces rapid T cell growth. In order to polarize T cells to a specific phenotype, the culture conditions are further refined [41].

CARs are encoded with viral vectors, which guide the RNA to reverse-transcribe into DNA and permanently integrate into the genome of the patient cells. The viral vector is washed out of the culture via medium exchange and/or dilution during the activation process. Lentiviral vectors are more commonly used than gammaretroviral vectors in clinical trials because of their safer integration site profile [42]. The other methods includes Sleeping Beauty transposon system and mRNA transfection. However, many concerns remain, such as the requirement for several rounds of infusion using transient mRNA transfection and the unknown potential of insertional mutagenesis and remobilization of transposons when using the Sleeping Beauty transposon system [43].

Three bioreactor culture systems are used to culture CAR-T cells: WAVE Bioreactor, G-Rex and CliniMACS Prodigy [44]. The major drawback of the former two systems is that the flask must be opened during cell inoculation. However, the CliniMACS Prodicy system is a single device that can effectively enrich, activate, transducer and expand the cells [45]. After the cells reach the numbers required for clinical uses, they are collected and transfused into the patients.

## Challenges for CAR-T cell therapy

Although CAR-T cells have been used in clinics, many questions still need to be addressed, such as the optimal vector and the long-term safety profile.

The key raw material for the CAR-T cell product is the viral vector that transduces the CARs into the T cells. The viral vector encoding the CARs can be made in large quantities and stored at −80 °C even for about 9 years [46]. Therefore, the sterility of the vector is crucial because these cells will be infused into the patients. The third-generation minimal lentiviral vector may be the safest one that has been developed thus far [47]. Quality control testing on the safety, sterility, titer, purity and potency is absolutely crucial. The comparison of vector purity, stability, and function is also important because vectors can be obtained from different suppliers. It would be most desirable to control these variables by using a single vector for the generation of CAR-T cells.

The potential for insertional mutagenesis caused by the integration of vector DNA into host cells is a concern. Lentiviral vectors may have a lower risk of mutagenesis than others [48]. However, both lentiviral and retroviral vectors are potentially oncogenic. The long-term safety of using viral vectors for CAR-T cell therapy remains unknown. Therefore, it is necessary to monitor carefully for any potential delayed adverse event related to these vectors for an extended period of time. The effects of persistent CAR-T cells on future pregnancies are also unknown.

Effective coordination among the collection, manufacturing, and treatment sites is crucial in order to ensure correct handling of the material and appropriate scheduling of patients during the therapeutic process. It is therefore very important to develop a standardized manufacturing process for CAR-T cells in order to establish the critical quality characteristics and target product profile. It is also important to gain more experience with these procedures because the CARs positivity, phenotype and viability vary among different products [49]. Comparing products is difficult because of the variability between the starting materials generated from leukapheresis.

The good results were observed in clinics with CAR-T cells therapy in hematological malignancies, however, it is still a change in the solid tumors because of antigen loss in tumor cells, the lack of unique antigens and the immune-suppressive tumor microenvironment of solid tumors [50, 51]. So in order to improve the effect of CAR-T cells therapy, many progresses have been made by incorporating CARs with another effector molecules such as PD1 switch receptors, anti-oxidant enzymes, matrix degradation enzymes and so on [52–56].

The major adverse effect observed after CAR-T cell therapy is severe cytokine release syndrome (CRS). Although most of the adverse events subsequent to CAR-T cell therapy can be managed with currently available interventions, it is nevertheless important to reduce the adverse effects while maintaining the efficacy of the treatment [57]. Similarly, it is also important to increase the safety of CAR-T cell therapy when improving the specificity of the modified T cells.

## Conclusion

CAR-T treatment for patients with tumors has shown promising outcomes; however, many remaining challenges need to be considered [58]. The high quality of CAR-T products needs to be ensured through optimization of protocols, and the long-term safety requires further study [59].

## Abbreviations

CARs: Chimeric antigen receptors
CAR-T cells: Chimeric antigen receptor redirected T cells
scFv: Single-chain Fragment variant
ITAMs: immunoreceptor tyrosine-based activation motifs
IL-2: Interleukin-2
TCR: T cell receptor
TRUCKs: T cell redirected for universal cytokine-mediated killing
APCs: Antigen-presenting cells
CRS: Cytokine release syndrome
